# Oversimplification risks too much: a response to ‘How predictable are mass extinction events?'

**DOI:** 10.1098/rsos.230400

**Published:** 2023-08-23

**Authors:** Carl J. Reddin, Martin Aberhan, Danijela Dimitrijević, Elizabeth M. Dowding, Ádám T. Kocsis, Gregor Mathes, Paulina S. Nätscher, Mark E. Patzkowsky, Wolfgang Kiessling

**Affiliations:** ^1^ Museum für Naturkunde, Leibniz Institute for Evolution and Biodiversity Science, Berlin, Germany; ^2^ GeoZentrum Nordbayern, Universität Erlangen-Nürnberg, Erlangen, Germany; ^3^ Paleontological Institute and Museum, University of Zurich, Zurich, Switzerland; ^4^ Department of Geosciences, Pennsylvania State University, University Park, PA, USA

## 1. Introduction

As anthropogenic climate change pushes modern ecosystems into uncharted territory, marine ectotherms show particularly clear climate change impacts [1,2]. Coincidentally, marine invertebrates have the lion's share of the macrofossil record, sometimes covering ancient episodes of rapid global warming (hyperthermals) that led to extinction crises, including mass extinctions [3]. This makes the fossil record an unrivalled but complex data resource. Foster *et al*. [4] present a rigorous comparison of three mass extinctions using a trait-based model and machine learning. Their main result, that the impacts of one mass extinction may not be a simple blueprint for the next mass extinction, has implications for the use of fossils to predict future extinction risk. However, we find their main conclusion, that ‘extinction selectivity during mass extinctions, therefore, appears to be unpredictable', to be only half of the story and a pessimistic oversimplification of their results. We discuss limitations to their analyses, especially where these highlight exciting avenues and alternative approaches to uncover and improve predictability of biotic crises using palaeobiology [5]. We conclude that the debate over whether the fossil record is a useful information source for extinction risk assessment is only just opening and emerging as a fruitful and urgent area of research.

### 1.1. Aim for events with modern-relevant drivers

Foster *et al*. [4] focus on three mass extinctions of which only two are thought to have comparable causes and rates of disturbance. Predictability scores between either the End-Permian or End-Triassic models (both hyperthermal events) and the End-Cretaceous (bolide impact) were the lowest among their comparisons [4]. Other studies (e.g. [6]) highlight how the fortunes of physiologically poorly buffered taxa changed from their selective extinction at both the End-Permian and End-Triassic to a selective extinction of physiologically buffered taxa at the End-Cretaceous. Evidence of shared environmental changes [3,7] and selectivity patterns (e.g. [6,8,9]) support shared extinction scenarios: global warming across the End-Permian and End-Triassic versus massive cooling and transient shutdown of primary production due to bolide impact for the End-Cretaceous. Cascading effects may indeed complicate attribution, creating dependences between functional group responses, but should be themselves targeted in future works.

### 1.2. Sampling heterogeneity

Uneven sampling in the fossil record is omnipresent and well known. Observed extinction records are often smeared back in time, more so for some organism groups than others, and the record of each mass extinction varies by the geography of its exposed outcrops (e.g. palaeolatitude) [10–12]. Such ‘confounders' in observational data may hide true relationships between features, or create fake ones [13,14]. Different sampling biases might overshadow true ecological similarities, or models may learn to predict shared sampling patterns rather than ecological impacts. Discerning hypothetical ecological signal from noise processes (e.g. sampling bias) is essential to decide the success of a model to predict or associate true ecological impacts (e.g. steps taken in [8]). Machine learning approaches are unfortunately no panacea [14].

### 1.3. Phylogenetic covariates

Phylogeny remains one of the strongest predictors of extinction [15] and covaries with organism trait grouping focused on by Foster *et al*. [4]. Groups such as ammonoids and rhynchonelliform brachiopods are generally more at risk of extinction than others [16], which may artificially inflate the importance of some of their traits. Contrasting mono-trait and poly-trait clades may help pick apart this chicken-and-egg puzzle (e.g. [8,15]).

### 1.4. Performance measurement

The benchmark for successful predictability depends on context and discipline. Foster *et al*. [4] use the standard threshold area under the curve (AUC) ≥ 0.7 as a good or acceptable model. The context needed here is that scores *within* an event only just meet this mark (AUC = 0.72, 0.72 and 0.8). These values should become the practical benchmark for the best between-event prediction that could be hoped for. If a model trained on random observations of extinction variation at an event struggles to provide a good prediction for extinction variation within the same event, then the conclusion must be that fossil data are difficult to predict from *per se*. The mean across the six between-event AUC values in Foster *et al*. [4] was 0.62, which, despite being far below perfect (i.e. AUC = 1), suggests predictable elements to the extinction signature. Identifying the predictable elements of extinction drivers, and the less predictable or less targeted elements (e.g. strongly biased by sampling) is an exciting avenue for future research (e.g. [8,9,17]).

### 1.5. Use modern ecological understanding to target specific impacts

Foster *et al*. [4] judged predictability based on overall extinction patterns, rather than targeting components hypothesized to be more predictable. Their approach is important given our limited understanding of what drives occurrence variation in the fossil record. However, it neglects the wealth of ecological understanding, which can provide a basis for hypotheses of palaeoecological responses under drivers of interest (e.g. [8,18,19] and references therein). Optimally, cause-effect mechanisms can be modelled to explore agreement between simulated and observed impacts (e.g. [9,17,20]).

### 1.6. Systems evolve

Evolutionary and Earth system changes are components we must account for in hypotheses of cause-effect, as Foster *et al*. [4] fittingly conclude but do not execute quantitatively. Quantitative appraisal indicates that some invertebrate groups changed their responses to hyperthermals more over time than others (e.g. sponges, organisms with calcitic skeletons), while reefs remain vulnerable [6,8]. This topic needs far more investigation, but evidence can be empirically targeted rather than raised theoretically.

## 2. Conclusion

We congratulate Foster *et al*. [4] on a sophisticated analysis that explores limits to the predictability we can expect from correlative models derived from the fossil record of mass extinctions. Armed with an ecological understanding of cause-effect mechanisms and their context-dependency [17,19], alongside a respect for biases in the fossil record [10,21] and the limitations of statistical models, we can be better informed when we cautiously approach the fossil record for hints at future perils. This is especially pressing in anticipation of a future mass extinction [22], and there are sufficient commonalities among warming-driven extinction events to state that the past is key to the future [5]. The fossil record of ancient crises, including mass extinctions, is an important, unique, but complex resource for developing our predictive capabilities. Oversimplifying its interpretation will only hinder this goal.

## Data Availability

No data are associated with this manuscript.
